# Anti-Tuberculosis Activity of *Pediococcus acidilactici* Isolated from Young Radish Kimchi against *Mycobacterium tuberculosis*

**DOI:** 10.4014/jmb.2107.07044

**Published:** 2021-09-28

**Authors:** Youjin Yoon, Hoonhee Seo, Sukyung Kim, Youngkyoung Lee, MD Abdur Rahim, Saebim Lee, Ho-Yeon Song

**Affiliations:** 1 Department of Microbiology and Immunology, School of Medicine, Soonchunhyang University, Cheonan 31151, Republic of Korea; 2 Probiotics Microbiome Convergence Center, Soonchunhyang University, Asan 31538, Republic of Korea

**Keywords:** *Mycobacterium tuberculosis*, *Pediococcus acidilactici*, probiotics, anti-tuberculosis effect, microbiome

## Abstract

Tuberculosis is a highly contagious disease caused by *Mycobacterium tuberculosis*. It affects about 10 million people each year and is still one of the leading causes of death worldwide. About 2 to 3 billion people (equivalent to 1 in 3 people in the world) are infected with latent tuberculosis. Moreover, as the number of multidrug-resistant, extensively drug-resistant, and totally drug-resistant strains of *M. tuberculosis* continues to increase, there is an urgent need to develop new anti-tuberculosis drugs that are different from existing drugs to combat antibiotic-resistant *M. tuberculosis*. Against this background, we aimed to develop new anti-tuberculosis drugs using probiotics. Here, we report the anti-tuberculosis effect of *Pediococcus acidilactici* PMC202 isolated from young radish kimchi, a traditional Korean fermented food. Under coculture conditions, PMC202 inhibited the growth of *M. tuberculosis*. In addition, PMC202 inhibited the growth of drug-sensitive and -resistant *M. tuberculosis*- infected macrophages at a concentration that did not show cytotoxicity and showed a synergistic effect with isoniazid. In a 2-week, repeated oral administration toxicity study using mice, PMC202 did not cause weight change or specific clinical symptoms. Furthermore, the results of 16S rRNA-based metagenomics analysis confirmed that dysbiosis was not induced in bronchoalveolar lavage fluid after oral administration of PMC202. The anti-tuberculosis effect of PMC202 was found to be related to the reduction of nitric oxide. Our findings indicate that PMC202 could be used as an anti-tuberculosis drug candidate with the potential to replace current chemicalbased drugs. However, more extensive toxicity, mechanism of action, and animal efficacy studies with clinical trials are needed.

## Introduction

*Mycobacterium tuberculosis*, the causative agent of human tuberculosis, is one of the most prevalent human pathogens. It infects a quarter of the world's population by developing sophisticated mechanisms to bypass the host’s innate and adaptive immune defenses [1]. Tuberculosis remains the leading cause of death from infectious diseases among adults worldwide [2]. According to a WHO report, in 2019, about 10 million people were infected with tuberculosis and about 1.4 million of them died from the disease [3]. Moreover, the pathogenic organism *M. tuberculosis* continues to evolve and increase its resistance to antagonists, leading to the development of multidrug-resistant (MDR) and extensively drug-resistant (XDR) *M. tuberculosis* strains, and ultimately, unmanageable totally drug-resistant (TDR) *M. tuberculosis* strains with even further developed resistance mechanisms [4]. It has been estimated that 3.7% of new cases of tuberculosis infection worldwide and 20% of previously treated cases are MDR tuberculosis [5], with a treatment success rate of only 54% [6]. As such, the development of a new anti-tuberculosis drug to solve the evolving tuberculosis problem is urgently needed.

Probiotics are defined as “live microorganisms which when administered in adequate amounts confer a health benefit on the host” that can be applied as single or multiple strains in a live or dead form in combination with prebiotics [7]. Probiotics can significantly affect the functions of the mucosal and systemic immune system through activation of several immune mechanisms. They are effective in controlling various diseases, including irritable bowel disease, allergies, diabetes, and cancer [8]. In addition to these diseases, probiotics have been extensively studied for controlling infectious diseases and pathogens [9] such as *Helicobacter pylori* [10], salmonellosis [11], candidal vulvovaginitis [12], urinary tract infection [13], *Clostridium difficile* [14], *Streptococcus pneumoniae* [15], and *Campylobacter* [16]. Moreover, research on infectious diseases and probiotics has recently been extended to antibiotic-resistant superbugs [17] such as vancomycin-resistant *Enterococcus* (VRE) [18], methicillin-resistant *Staphylococcus aureus* (MRSA) [19], carbapenem-resistant *Enterobacteriaceae* (CRE) [20], MDR *Pseudomonas aeruginosa* [21], and viral infections such as human immunodeficiency virus (HIV) [22] and severe acute respiratory syndrome coronavirus 2 (SARS-CoV-2) [23].

As mentioned above, there have been many reports of the preventive and therapeutic effects of probiotics on various infectious diseases. However, research on the effects of probiotics against tuberculosis is still lacking, and therefore we investigated probiotics with the goal of discovering potential anti-tuberculosis drug candidates. In this study, we report the anti-tuberculosis effect of *Pediococcus acidilatici* PMC202 isolated from traditional Korean fermented foods.

## Materials and Methods

### Isolation of Probiotic Strain from Traditional Fermented Foods

The probiotic strain was isolated from young radish kimchi, a Korean traditional fermented food, in the laboratory of Soonchunhyang University in 2018. The liquid portion of each sample was streaked onto a plate of MRS agar (de Man, Rogosa and Sharp, BD Difco, USA) using a loop. The plates were cultured in an aerobic incubator (general incubator, N-Biotek, Korea) at 37°C. The colonies were then cultured in MRS broth (BD Difco) and stored at -80°C in 15% glycerol stock. Identification was done by 16S rRNA gene sequencing.

### 16S rRNA Gene Sequencing of the Probiotic Strain

The 16S rRNA gene sequencing was performed by Biofact (Korea). Briefly, DNA was extracted by repeated heating and ice-cooling. Primers 27F (5'-AGA GTT TGA TCC TGG CTC AG-3') and 1492R (5'-GGT TAC CTT GTT ACG ACT T-3') were used for PCR on a Hushrun PCR cycler (Biofact, Korea). The amplified PCR product was purified and sequenced using an ABI PRISM 3730XL DNA analyzer (Applied Biosystems, USA) using a BigDye Terminator v3.1 Cycle Sequencing Kit (Thermo Fisher Scientific, USA). Sequences were compared with the National Center for Biotechnology Information (NCBI) GenBank database using BLAST (basic local alignment search tool).

### Preparation of Probiotic Strain Extract

All probiotic strains were cultured in 30 ml of MRS broth (BD Difco) and incubated at 37°C in a shaking incubator (BioFree, Korea) for 24 h. Cultures were adjusted to an OD_600nm_ value of 1.0 using a spectrophotometer (DR 1900, Hatch, USA). After that, centrifugation was performed at 4,000 g for 10 min using a centrifuge (Combi R515, Hanil Scientific, Inc., Korea), followed by washing with 0.85% NaCl solution to remove medium components. This process was repeated three times to collect the pellet. After adding 1 ml of 0.85% NaCl solution to the pellet followed by vortexing, it was transferred to a Lysing Matrix B tube (MP Biomedicals, USA) containing 0.1 mm silica spheres and disrupted for 1 min using a homogenizer (FastPrep-24 5G, MP Biomedicals). The probiotic extract was then boiled for 10 min prior to anti-mycobacterial testing.

### *M. tuberculosis* Strain and Culture Conditions

*M. tuberculosis* H37Rv (ATCC 27294) was purchased from the American Type Culture Collection (ATCC, USA), and XDR *M. tuberculosis* (KMRC 00203-00197) was obtained from the Korean Mycobacterium Resource Center (KMRC, Korea). Based on the microdilution technique, the concentrations of isoniazid (INH), rifampicin (RIF), streptomycin (STR), pyrazinamide (PZA), and ethambutol (EMB) were 0.19, 0.09, 0.19, >200, and 0.78 μg/ml for *M. tuberculosis* H37Rv, respectively, and 25, 100, 1.56, >200, and 6.25 μg/ml for XDR *M. tuberculosis*, respectively [24]. *M. tuberculosis* was cultured at 37°C in Middlebrook 7H9 broth (BD Difco) supplemented with 10% OADC (oleic acid-albumin-dextrose-catalase) (BD Difco) and 0.5% Tween 80 (Sigma-Aldrich, USA). All experiments using *M. tuberculosis* were conducted in an Animal Biosafety Level 3 Laboratory of Soonchunhyang University (ABSL-3, KDCA-20-3-04).

### Cell Line and Culture Conditions

The murine macrophage RAW 264.7 cell line (KCLB 40071) was purchased from the Korean Cell Line Bank (KCLB, Korea). The cells were prepared in Dulbeccós Modified Eagle Medium (DMEM, Gibco) supplemented with 10% fetal bovine serum (FBS) (Gibco) and 1% antibiotics (100 U/ml penicillin and 100 μg/ml streptomycin)(HyClone, USA) followed by incubation at 37°C in a 5% CO_2_ atmosphere.

### Intracellular Anti-Mycobacterial Activity Test with Ziehl-Neelsen Staining

RAW 264.7 cells (1 × 10^5^ cells/ml) grown for 24 h in a 5% CO_2_ incubator were seeded onto 2-well cell culture slides (SPL Life Sciences, Korea) until confluence reached approximately 70-80%. Cells were then exposed to H37Rv or XDR *M. tuberculosis* strains at a multiplicity of infection (MOI) of 10:1 for 2 h to induce intracellular infection. After washing the cells three times with 1× phosphate-buffered saline (PBS), 2 ml of DMEM without antibiotics containing various concentrations of probiotic strain extract was added to each well and incubated for 3 days at 37°C with a 5% CO_2_ atmosphere. Cells were then washed three times with 1× PBS to remove residues. After Ziehl-Neelsen staining, the cells were observed with an optical microscope (AX10, Carl Zeiss, Germany) at 1,000× magnification.

### Intracellular Anti-Mycobacterial Activity Test Using CFU Assay

The intracellular anti-mycobacterial activity test was similar to the test for intracellular anti-tuberculosis effect using Ziehl-Neelsen staining. In this test, 96-well plates were used. The volume of each well was 200 μl. The colony-forming unit (CFU) method was used instead of staining to measure the anti-mycobacterial effect. Other conditions such as cultured cell types, cell culture/density, and infection conditions were the same. After 3 days of incubation, the cells were lysed with distilled water (DW) on the principle of osmotic pressure. Dilutions (10-fold) were spread onto 7H10 agar medium (BD Difco) plates. The *M. tuberculosis* CFU counts were then determined one month later.

### Anti-Mycobacterial Activity in Coculture Conditions

The in vitro anti-tuberculosis activity of the probiotic was tested by coculturing the probiotic strain (2 × 10^6^ CFU/ml) and *M. tuberculosis* H37Rv (2 × 10^8^ CFU/ml). The broth medium used consisted of 10% MRS broth and 90% 7H9 broth. Both strains were cultured for two weeks in an incubator at 37°C with shaking (180 rpm). On days 0, 3, 6, 9, and 12, the CFUs of *M. tuberculosis* were counted. At the same time, the acidity was measured using a pH meter. Conditions wherein the initial pH was adjusted to 5 or 6.8 using hydrochloric acid (Sigma-Aldrich) or sodium hydroxide (Sigma-Aldrich) were also analyzed.

### Cell Cytotoxicity

To evaluate the cytotoxicity of the probiotic, trypan blue and methylene blue staining were performed. Briefly, RAW 264.7 cells were seeded onto 2-well cell culture slides at a density of 1 × 10^5^ cells/ml and then incubated at 37°C with a 5% CO_2_ atmosphere for 24 h until confluency reached about 70-80%. After incubation, cells were washed with 1× PBS and then incubated at 37°C in a 5% CO_2_ atmosphere for 3 days with probiotic extract. Cells were observed using an optical microscope (AX10, Carl Zeiss) after staining with methylene blue (Dagatron, Korea). The number of viable cells was measured with a hemocytometer (Marienfeld, Germany) after cells detached with a scraper were stained with trypan blue (Gibco, USA).

### Repeated-Dose Toxicity in Mice by Oral Administration

Six-week-old female Balb/C mice were obtained from Koatech (Korea). Upon arrival, all animals were inspected for health status to confirm suitability for study. The mice were acclimatized to the laboratory environment for 7 days, housed (6 per cage) in an environment-controlled barrier animal room, and given free access to a standard commercial diet and drinking water *ad libitum*. All animal rooms were monitored and maintained under a 12-h light/dark cycle (150-300 Lux) at a temperature of 19-25°C and 30-70% relative humidity.

The probiotic strain was inoculated into MRS broth at 0.1%, incubated at 37°C for 24 h, and washed with 0.85%NaCl solution. The probiotic was then adjusted to 6 × 10^8^ CFU/ml, of which 200 μl was orally administered once daily, five times a week, for a total of two weeks using a zonde. The control group was administered with a 0.85%NaCl solution without probiotics. Acute toxicity was assessed based on clinical signs, body weight, and mortality within the dosing period. At the end of the experiment, lungs were removed, and bronchoalveolar lavage (BAL) fluid was collected in the same way as previously reported [25].

This animal experiment was conducted at Soonchunhyang University's PMC Animal Lab, which is registered as an animal testing facility (KFDA 657) in accordance with the regulations of the Act on Laboratory Animals licensed as ABSL-2 (LML 20-591). The animal experimentation plan in this study was reviewed and approved by the Institutional Animal Care and Use Committee (IACUC) of Soonchunhyang University (Approval No. 2021-0047).

### Metagenomics Analysis of BAL Fluids

According to the manufacturer's instructions, total DNAs were extracted from BAL samples using a QIAamp DNA Mini Kit (Qiagen). Next, the V4 hypervariable region was amplified using a primer set (Forward primer: TCGTCGGCAGCGTCAGATGTGTATAAGAGACAG-CCTACGGGNGGCWGCAG, Reverse primer: GTC TCGTGGGCTCGGAGATGTGTATAAGAGACAG-GACTACHVGGGTATCTAATCC) capable of amplifying the primer sequences 515f to 806r of the 16S rRNA gene. The part before the dash in the primer was the overhang adapter sequence required later for indexing. The part after the dash in the primer was a locus-specific sequence to obtain a product of 359 bp after the first PCR. Using a primary PCR product as a substrate, a metagenomic library was prepared using a Nextera XT DNA Library Prep Kit (Illumina, USA). For PCR, 2×KAPA HiFi HotStart ReadyMix (Kapa Biosystems, USA) was used. After each step, AMPure XP beads (Beckman Coulter, UK) were used for cleanup. The concentration and quality thereof were then checked. The sample was finally diluted from 1 nM to 50 pM with 10 mM Tris (pH 8.5). After the addition of a 10% PhiX Control Library (Illumina), the library sample was loaded into an iSeq-100 reagent cartridge (Illumina). After sequencing with an iSeq-100 platform (Illumina), the sequencing data were analyzed using the EzBioCloud server (Cheonlab, Korea).

### Quantification of Nitric Oxide

The concentration of nitrite (NO_2_^-^), which is used as an indicator of nitric oxide (NO) synthesis, was measured using Griess reagent as previously reported [26]. Briefly, RAW 264.7 cells were seeded into 96-well cell culture plates at a density of 1 × 10^5^ cells/ml per well, cultured for 24 h at 37°C, and then infected with *M. tuberculosis*. After washing three times with 1× PBS, the cells were treated with probiotic extract for 3 days. For NO quantification, 50 μl of the cell culture supernatant was transferred to a new 96-well plate, mixed with the same amount of Griess reagent solution (G2930, Promega, USA), and incubated at room temperature for 10 min. The absorbance was then measured at 540 nm with a microplate reader (Victor Nivo, Perkin-Elmer, USA).

### Whole-Genome Sequencing of Probiotic Strain

The probiotic strain was inoculated into MRS broth at a ratio of 0.1%. Cells were obtained at the late exponential phase of growth. After washing three times with PBS, gDNA was extracted using a QIAamp DNA Mini Kit (Qiagen). PacBio library construction and whole-genome sequencing were performed by Chunlab. Genomic DNA was cut into 10 kb using a g-tube (Covaris, USA) and purified. Ends were repaired, and SMRTbell adapters were ligated to the blunt end using the SMRTbell Template Prep Kit 1.0 (PacBio, USA). The library was then sequenced using PacBio P6C4 chemistry in an 8-well-SMART Cell v3 of PacBio RSII (PacBio). PacBio sequencing data were assembled with PacBio SMRT Analysis 2.3.0 using the HGAP2 protocol. The genome was then circularized using a Circlator 1.4.0 (Sanger Institute, UK). Protein coding sequences (CDSs) were predicted with Prodigal 2.6.2 [27] and grouped according to roles regarding orthologous groups (EggNOG; http://eggnogdb.embl.de). Genes encoding tRNAs were searched using tRNAscan-SE 1.3.1 [28]. rRNAs and other noncoding RNAs were searched by covariance model searches using the Rfam 12.0 database [29]. For comparison of prokaryotic genome sequences, OrthoANIu algorithm-based Average Nucleotide Identity (ANI) calculator (https://www.ezbiocloud.net/tools/ani) was used [30].

## Results

### 16S rRNA Gene Sequencing-Based Identification of Isolated Probiotic

The probiotic strain isolated from young radish kimchi was taxonomically identified through 16S rRNA gene sequencing. This probiotic strain’s 16S rRNA gene sequence was compared with sequences deposited in the NCBI reference sequence database. The analysis result showed that the sequence of the strain was 99% similar to 16S rRNA sequences of *P. acidilactici* strains DSM 20284 and NGRI 0510Q. In addition, the sequence was similar (97%to 98%) to those of strains *P. pentosaceus* DSM20336, *P. stilesii* FAIR-E 180, *P. claussenii* ATCC BAA-344, and *P. argentinicus* CRL 776, all belonging to genus *Pediococcus* (Table 1). These results indicate that the isolated strain could be a species in the genus *Pediococcus*.

### Whole-Genome Analysis Result of the Strain

**Fig. 1:**
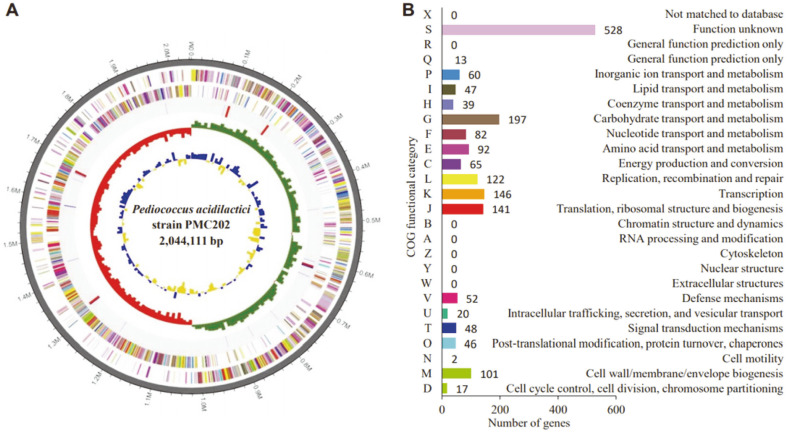
High-throughput genome sequencing results of *P. acidilactici* strain PMC202. (**A**) The genome of the *P. acidlactici* PMC202 strain is shown as a circular map. Antisense/sense strands are colored according to the cluster of orthologous gene (COG) category. tRNA and rRNA are shown in red and blue from the periphery to the center. The inner-circle represents GC skew, with yellow and blue representing positive and negative values, respectively. GC content is shown in red and green. (**B**) Relative abundance by COG functional category cluster of genes is shown.

The whole-genome sequencing analysis result of the strain is shown in Fig. 1. The genome consists of 2,044,111 bp single circular chromosomes with 1,954 coding DNA sequences (CDSs) (Fig. 1A). A total of 1,929 proteins of predicted CDS were functionally classified according to the Clusters of Orthologous Groups (COGs) (Fig. 1B). Biological functions could be defined for 1,401 predicted proteins, while 528 CDS were homologous to conserved proteins with unknown functions in other organisms. The other 25 hypothetical proteins did not match with any known proteins in the database.

### OrthoANI Genomic Similarity

**Fig. 2:**
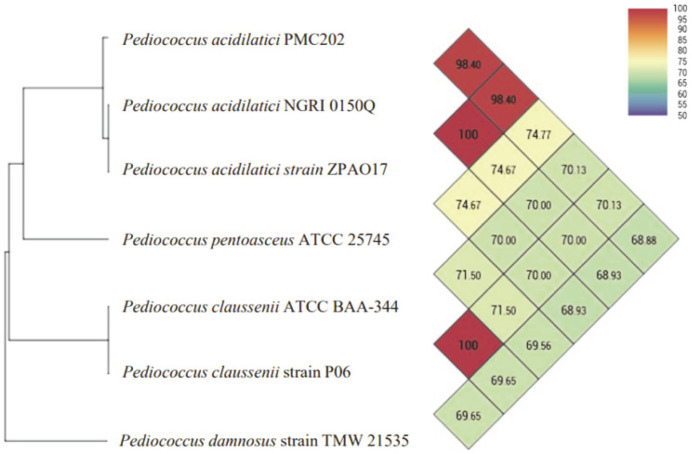
OrthoANI results calculated from available genomes of *Pediococcus* species. The OrthoANI value of *P. acidilatici* PMC 202 and *P. acidilatici* NGRU 0150Q was 98.40, higher than 96.0%, the standard for determining the same species.

Similarity analysis was performed using the OrthoANI method for strains that shared high similarities in the 16S rRNA analysis using the entire genome sequence data of the strain (Fig. 2). The analysis result confirmed that its similarity with a strain (ZPA017, NGRI 0510Q) of *P. acidilactici* was 98.40%. Its similarities with other species of the *Pediococcus* genus such as *P. pentosaceus*, *P. claussenii*, and *P. damnosus* were 74.77%, 70.13%, and 70.13%, respectively. As a result, it was confirmed that the newly discovered strain was a *P. acidilactici* species of the *Pediococcus* genus.

### Comparison of Genomic Characteristics with Different Strains of *P. acidilactici* Species

The genome of PMC202 was then compared with published genome information on other strains of *P. acidilactici* (PMC48, K3, S1, JQII-5, HN9) (Table 2). The PMC 202 strain differed from other *P. acidilactici* strains in source, genome size, G+C content, CDS, and rRNA/tRNA numbers. Thus, the PMC202 strain was judged as a new strain different from existing strains.

### Intracellular Anti-Mycobacterial Activity of PMC202

**Fig. 3:**
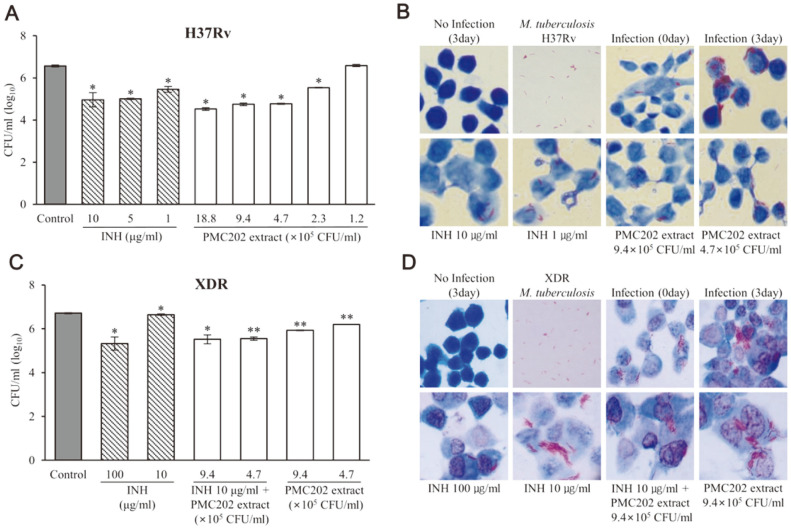
Effect of PMC202 on *M. tuberculosis*-infected macrophages. Macrophages were infected with (**A, B**) *M. tuberculosis* H37Rv or (**C, D**) XDR *M. tuberculosis*, treated with heat-treated PMC202 extract for 3 days. (**A, C**) CFU quantification results, (**B, D**) Ziehl-Neelsen staining images. The experiment was performed three times in triplicate. Values are expressed as mean values and standard deviations (SD). Statistical significance with controls was analyzed using unpaired Student's *t*-test. *p < 0.05; **p < 0.01.

The inhibitory effect of PMC202 on *M. tuberculosis* in macrophages was tested (Fig. 3). RAW 264.7 cells were infected with (A, B) *M. tuberculosis* H37Rv or (C, D) XDR *M. tuberculosis*, treated with heat-treated PMC202 extract for 3 days, and analyzed by (A, C) CFU method or (B, D) Ziehl-Neelsen staining method. Compared with the untreated control sample, PMC202 at 2.3 × 10^5^ CFU/ml, 4.7×10^5^ CFU/ml, 9.4×10^5^ CFU/ml, and 18.8 × 10^5^ CFU/ml significantly inhibited *M. tuberculosis* H37Rv. This effect was similar to INH at 1 μg/ml or 5 μg/ml (Fig. 3A). This anti-mycobacterial effect was also confirmed through staining. It was found that purple-colored *M. tuberculosis* increased at three days after infecting macrophages with *M. tuberculosis* (Fig. 3B). However, there was a relatively small amount of *M. tuberculosis* in samples treated with PMC202 or INH.

Unlike results for *M. tuberculosis* H37Rv, 10 μg/ml of INH treatment had no significant anti-mycobacterial effect on XDR *M. tuberculosis* (Fig. 3C). However, the effect of PMC202 on XDR *M. tuberculosis* was similar to that on *M. tuberculosis* H37Rv after treatment at 4.7 × 10^5^ CFU/ml and 9.4 × 10^5^ CFU/ml. In particular, for samples treated with 10 μg/ml of INH and 4.7 × 10^5^ CFU/ml or 9.4 × 10^5^ CFU/ml of PMC202 simultaneously, XDR *M. tuberculosis* was reduced more than that in samples treated with each alone. The anti-mycobacterial effect of PMC202 on XDR *M. tuberculosis* was also confirmed through the staining method (Fig. 3D).

### Anti-Tuberculosis Activity of PMC202 in Broth Coculture Condition

**Fig. 4:**
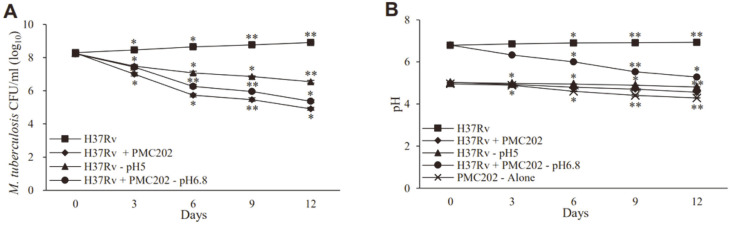
Anti-mycobacterial activity of PMC202 against *M. tuberculosis* H37Rv in coculture conditions. (**A**) The CFU of *M. tuberculosis* and (**B**) the pH of the culture were measured on days 0, 3, 6, 9, and 12 while culturing only *M. tuberculosis* (square) and coculturing *M. tuberculosis* and PMC202 (rhombus). The initial inoculation density was 2 × 10^8^ CFU/ml for *M. tuberculosis*, 2 × 10^6^ CFU/ml for PMC202, and was cultured at 37°C 180 rpm in 10 ml of 7H9 broth containing 10% MRS broth. The initial pH of culturing *M. tuberculosis* alone and coculture conditions with *M. tuberculosis* and PMC202 were 6.8 and 5.0, respectively, and as time passed, both CFU and pH of *M. tuberculosis* increased in the former case and decreased in the latter case. In addition, the conditions of culturing only *M. tuberculosis* adjusted to an initial pH of 5.0 (triangle), coculture adjusted to an initial pH of 6.8 (circle), and culturing only PMC202 without *M. tuberculosis* (cross) were also measured. Experiments were performed three times in triplicate, and values are expressed as mean and SD. Statistical significance with initial value was analyzed using unpaired Student's *t*-test. *p < 0.05; **p < 0.01.

The ability of PMC202 to inhibit *M. tuberculosis* H37Rv was evaluated in broth coculture conditions (Fig. 4). The CFU of *M. tuberculosis* (Fig. 4A) and the broth’s pH (Fig. 4B) were measured on days 0, 3, 6, 9, and 12 while culturing *M. tuberculosis* alone or in a coculture with PMC202. The initial pH of *M. tuberculosis* single culture and coculture with PMC202 were 6.8 and 5.0, respectively, and after 12 days, the former increased to 8.1 × 10^8^ CFU/ml and pH 7.0, and the latter decreased to 8.7 × 104 CFU/ml and pH 4.5. In addition, after 12 days of incubation, the culture of *M. tuberculosis* adjusted to the initial pH of 5 became 3.5 × 10^6^ CFU/ml and pH 4.81, and the coculture adjusted to the initial pH of 6.8 became 2.4 × 10^5^ CFU/ml and pH 5.28, and this decrease was greater than in the single culture. When PMC202 was cultured alone without *M. tuberculosis*, the pH gradually decreased during the incubation period and finally decreased to 4.3.

### Cytotoxicity of PMC202

**Fig. 5:**
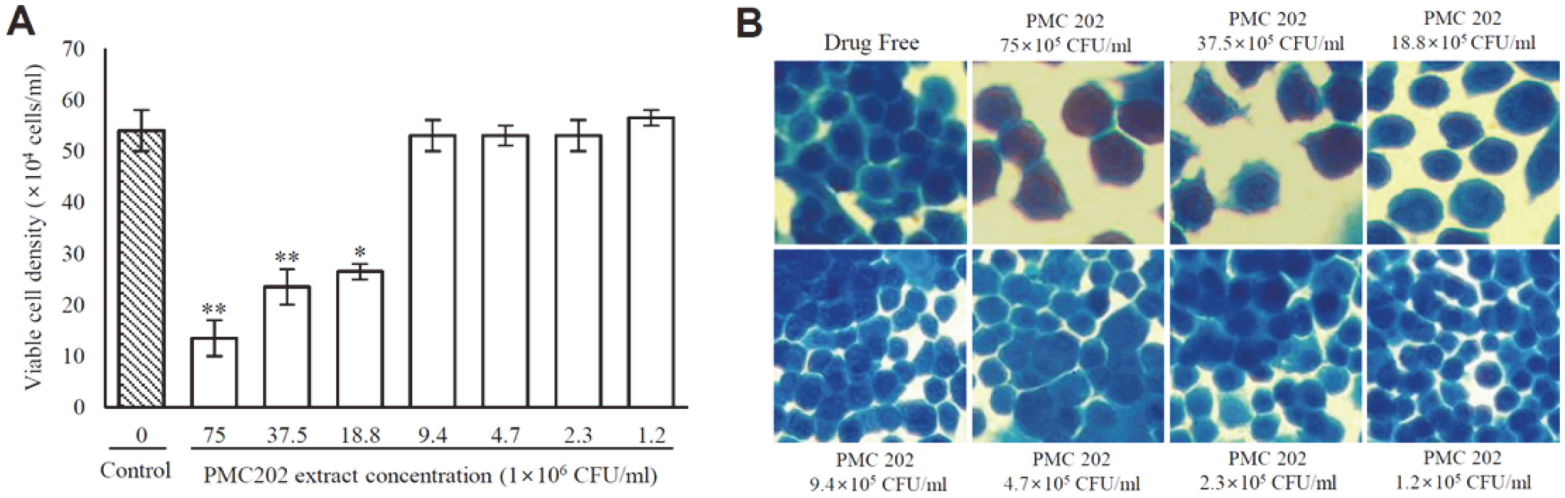
Cytotoxicity evaluation of PMC202 using RAW 264.7 cells. RAW 264.7 cells were treated with heat-treated PMC202 extract at different concentrations for 3 days, (**A**) Live cells were counted with a hemocytometer after trypan blue staining, (**B**) Toxicity to cells was evaluated by methylene staining. The experiment was performed in triplicate three times. Values are expressed as mean ± SD. Statistical significance vs. probiotics-free control was determined using unpaired Student's *t*-test. *p < 0.05; **p < 0.01.

The cytotoxicity of PMC202 extract to RAW 264.7 cells was evaluated (Fig. 5). The trypan blue staining test result showed that PMC202 at 9.4 × 10^5^ CFU/ml or less did not affect the viability of macrophages. However, the viability of macrophages was significantly reduced when they were treated with PMC202 at a concentration higher than 18.8 × 10^5^ CFU/ml (Fig. 5A). When cells were stained with methylene blue and observed under an optical microscope, cytotoxicity was observed when PMC202 at 18.8 × 10^5^ CFU/ml or more was used for treatment, similar to the results of the trypan blue method (Fig. 5B).

### Repeated Oral Toxicity Assay of PMC202 in Mice

**Fig. 6:**
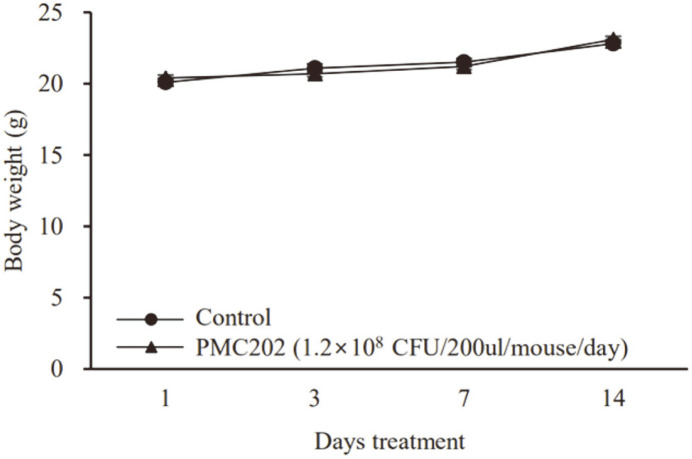
Body weight of mice during two weeks of repeated oral administration of PMC202. All mice were weighed on days 1, 3, 7, and 14 while the freshly prepared live PMC202 strain was orally administered at 1.2 × 10^8^ CFU per mouse once a day, five days a week, for two weeks.

Acute toxicity was investigated after mice were repeatedly treated with PMC202 through oral administration for two weeks (Fig. 6, Table 3). As a result, there was no significant change in body weight for mice administered with PMC202 compared to the group of mice administered with 0.85% NaCl solution (Fig. 6). Death and unusual clinical changes were not observed after PMC202 administration (Table 3).

### Analysis of Microbiome Changes in BAL Fluid After Oral Administration of PMC202 to Mice

**Fig. 7:**
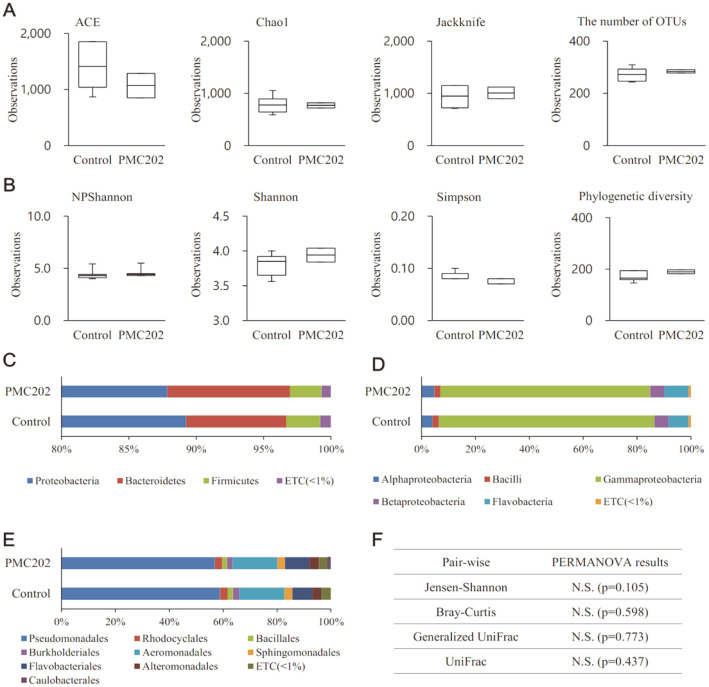
Analysis of effects of PMC202 on lung microbiome in mice. Alpha-diversity was measured through (**A**) species richness and (**B**) diversity index. Averaged taxonomic compositions between two groups were compared at (**C**) phylum, (**D**) class, or (**E**) order level. (**F**) Beta-diversity was evaluated through beta set-significance analysis. Taxonomic relative abundance was analyzed using the In Wilcoxon rank-sum test. N.S., Not significant.

The microbial community change in BAL fluid after administration of PMC202 was analyzed through a metagenomic analysis based on next-generation sequencing (NGS) technology (Fig. 7). After analyzing all of the applied statistical techniques, we confirmed that PMC202 administration did not cause a significant change in species richness (Fig. 7A) or diversity index (Fig. 7B). In the case of the averaged taxonomic composition at the phylum (Fig. 7C), class (Fig. 7D), or order (Fig. 7E) level, there was no significant difference among taxa having a composition of 1% or more. Moreover, beta-diversity analysis showed there was no significant difference in microbiome community between the two groups (Fig. 7F).

### Evaluation of the Effect of PMC202 on NO Production

**Fig. 8:**
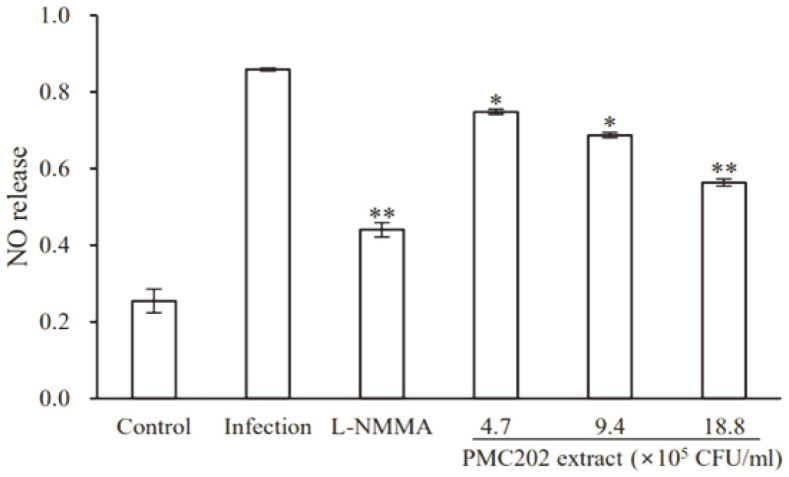
Effects of PMC202 on nitrites (NO_2_^-^) in *M. tuberculosis*-infected macrophages. After infecting RAW 264.7 cells with *M. tuberculosis* H37Rv, heat-treated PMC202 extract was used for treatment for three days. As a nitric oxide (NO) indicator, nitrite was quantified using a Griess reagent. L-NG-mono-methylarginine (L-NMMA) was used as a negative control. Experiments were performed three times in triplicate. Values are expressed as mean ± SD. Statistical significance vs. probiotic-free controls was determined using unpaired Student's *t*-test. *p < 0.05; **p < 0.01.

The effect of PMC202 on the production of NO was tested (Fig. 8). RAW 264.7 cells were infected with *M. tuberculosis* H37Rv (Fig. 8) and treated with PMC202 for 3 days. Griess reagent for quantifying nitrite as a NO indicator was then used. Nitrite production was induced in RAW 264.7 cells at 3 days after infection with *M. tuberculosis*. PMC202 at 4.7 × 10^5^ CFU/ml, 9.4×10^5^ CFU/ml, and 18.8 × 10^5^ CFU/ml reduced nitrite production by 12.1%, 15.2%, and 18.3% in *M. tuberculosis* H37Rv-infected macrophages.

## Discussion

From mono-drug-resistant to MDR, XDR, and most recently TDR, the rapid evolution of *M. tuberculosis* will continue to make tuberculosis an even more incurable disease unless new treatment options are soon available [31]. To manage drug-resistant tuberculosis, a variety of potential strategies are being proposed, including the use of a pathogen-centric approach of developing new compounds with different mechanisms of action, repurposing drugs, using new analogues of existing anti-tuberculosis drugs, and using host-centric approaches of immunomodulators, therapeutic vaccines, immunity, and cell therapy [32]. As a form of alternative treatment, probiotics have recently been highlighted for their potential roles in controlling tuberculosis [33]. In this study, probiotics were applied to develop an alternative approach to solve the problem of antibiotic-resistant *M. tuberculosis*, and we have reported the anti-tuberculosis effect of *P. acidilactici* PMC202 isolated from Korean traditional fermented food.

PMC202, a bacterium isolated from young radish kimchi, was judged to be *P. acidilactici* according to similarity cutoff criteria of 98.65% based on 16S gene sequencing [34] and 95% based on the whole genome [35]. In addition, PMC202 was determined to be a novel strain because its source and genetic characteristics were different from other strains of the *P. acidilactici* species.

PMC202 showed a significant anti-mycobacterial effect in the coculture experiment with *M. tuberculosis* H37Rv. This result was similar to the result of *Lactobacillus* reducing the number of *M. bovis* in coculture conditions. This effect was related to the pH decrease due to the organic acid production of lactic acid bacteria. Thus, despite its limitations as a pulmonary tuberculosis model, it might be suitable as an in vitro model of intestinal tuberculosis, which is known to account for 3 to 5% of extrapulmonary tuberculosis cases [36].

Tuberculosis infection of the host begins after inhalation of an aerosol containing a small number of bacilli [37]. Once entering the lungs, these bacilli are internalized through phagocytosis by alveolar macrophages [37]. RAW 264.7 macrophages are used as a general cell model in tuberculosis research [38], and were therefore used as an in vitro model in the present study. The intracellular anti-mycobacterial effect of PMC202 was then investigated. PMC202 showed an effect at a concentration that did not show cytotoxicity against drug-sensitive and -resistant *M. tuberculosis*. In particular, it also showed a synergistic effect with INH against XDR *M. tuberculosis*. These results indicate that PMC202 can be used as an adjuvant in conjunction with standard chemotherapy to treat *M. tuberculosis* infection.

Although currently available drugs are effective in treating tuberculosis disease or latent infection, they can cause serious side effects [39]. Drug-resistant tuberculosis, in particular, is treated with therapies that include second-line drugs with relatively high side effects, even death [40]. Therefore, toxicity evaluation is very important for newly developed anti-tuberculosis drugs. In this study, *P. acidilactici* was developed as an anti-tuberculosis drug candidate, is generally recognized as safe (GRAS), and has probiotic properties such as beneficial enzyme activity [41]. Thus, it is widely used in the fermentation of food and starter culture for cheese and yogurt production [42].

Moreover, this strain was isolated from traditional fermented foods and is considered to be relatively safe. Despite this, there have been reports of toxicity and sepsis, especially in immunocompromised patients, even for strains well known as probiotics [43]. Therefore, a repeated oral administration toxicity test was conducted using mice for two weeks in this study. As a result, weight change, death, and specific clinical symptoms were not observed.

The microbiota that inhabits the body can modulate several endocrinal, neuronal, and immune pathways in the host, thus affecting essential human functions, including digestion, energy metabolism, and inflammation [44]. Antibiotic treatment can cause changes in the microbiome, depending on the type of antibiotic, dose, and duration of exposure. This dysbiosis is closely related to disease and health [45]. The WHO guidelines recommend 6 months of multi-drug therapy for new pulmonary tuberculosis patients [46]. However, patients with MDR-tuberculosis require high-dose chemotherapy with a second-line drug for 9 to 24 months [47]. There is a growing interest in the relationship between tuberculosis chemotherapy, which requires a high-dose combined antibiotic therapy over a long period of induction known to destroy the human microbiome and its side effects [48]. In recent years, the profound impact of anti-tuberculosis therapy on the composition of the lung microbiome, which plays a role in pathophysiological processes associated with tuberculosis disease, has become increasingly important [49]. Therefore, a metagenomic analysis based on the 16S rRNA gene was performed for mouse BAL samples to evaluate the effect of oral administration of PMC202 on changes in the lung microbiome. As a result, PMC202 did not significantly affect species richness, species diversity, or taxonomic composition. It did not induce significant differences in microbial communities either.

Probiotics can regulate the innate/acquired immune system by influencing the mucosal/systemic immune response; thus, they are applied as immunotherapy [50]. From this perspective, the importance of the potential role of probiotics in the treatment of tuberculosis has been highlighted [33]. As such, the inhibitory effect of PMC202 on *M. tuberculosis* in macrophages seems to be related to the regulation of immune response. Therefore, we analyzed its association with NO, which is known to play a versatile role in the immune system [51]. The analysis showed that NO levels increased by *M. tuberculosis* infection were decreased by PMC202. This phenomenon can be interpreted several ways. As previously reported, it seems to be related to the cytoprotective effect [52]. Probiotics have been proposed to mediate immune responses by activating several inflammatory cytokines and interleukins associated with tuberculosis, but considering the lack of sufficient research, further studies are needed to elucidate the relevant mechanisms [33, 53].

In summary, this study showed the effects of *P. acidlactici* PMC202 newly isolated from young radish kimchi on *M. tuberculosis* in macrophages and suggested that it could be used as a candidate anti-tuberculosis agent for treating drug-resistant tuberculosis. However, more extensive studies, including evaluation of the in vivo animal efficacy of PMC202, clinical trials, and its mechanism of action, are needed. These findings highlight the potential role of using probiotics as a novel strategy in the treatment of tuberculosis.

## Figures and Tables

**Table 1:** 16S rRNA gene sequence analysis and blast analysis results with relevant data from NCBI.

NCBI reference	Organism	Length	Score	Identities	Gaps
NR_042057.1	*Pediococcus acidilactici* DSM 20284	1569	2724 bits (1475)	1496/1505 (99%)	5/1505 (0%)
NR_041640.1	*Pediococcus acidilactici* NGRI 0510Q	1516	2695 bits (1459)	1467/1470 (99%)	3/1470 (0%)
NR_042058.1	*Pediococcus pentosaceus strain* DSM 20336	1569	2612 bits (1419)	1478/1506 (98%)	5/1506 (0%)
NR_042401.1	*Pediococcus stilesii* strain FAIR-E 180	1529	2564 bits (1388)	1460/1496 (98%)	3/1496 (0%)
NR_075029.1	*Pediococcus claussenii* strain ATCC BAA-344	1567	2510 bits (1359)	1459/1507 (97%)	7/1507 (0%)
NR_042623.1	*Pediococcus argentinicus* strain CRL 776	1492	2477 bits (1341)	1444/1494 (97%)	7/1494 (0%)
NR_042232.1	*Pediococcus claussenii* strain P06	1472	2418 bits (1309)	1418/1471 (96%)	6/1471 (0%)
NR_029136.1	*Pediococcus parvulus* strain S-182	1436	2302 bits (1246)	1374/1437 (96%)	6/1437 (0%)
NR_043291.1	*Pediococcus ethanolidurans* strain Z-9	1501	2344 bits (1269)	1404/1470 (96%)	6/1470 (0%)
NR_113922.1	*Pediococcus parvulus* strain NBRC 100673	1501	2381 bits (1289)	1428/1496 (95%)	8/1496 (1%)
NR_043290.1	*Pediococcus cellicola* strain Z-8	1542	2372 bits (1284)	1431/1503 (95%)	6/1503 (0%)
NR_025388.1	*Pediococcus inopinatus* strain DSM 20285	1551	2361 bits (1278)	1429/1503 (95%)	6/1503 (0%)
NR_025388.1	*Pediococcus inopinatus* strain DSM 20285	1551	2361 bits (1278)	1429/1503 (95%)	6/1503 (0%)
NR_042087.1 *Pediococcus damnosus* strain DSM 20331 1561 2344 bits (1269) 1427/1503 (95%) 11/1503 (1%)

NCBI, National Center for Biotechnology Information.

**Table 2:** Comparison of chromosomal properties of *P. acidilactici* strains.

Strain	PMC202	PMC48	K3	S1	JQII-5	HN9
Sources	Young radish	Sesame leaf	Nuruk	Makgeolli	Fermented dairy	Pork sausage
	kimchi	kimchi				
Genome size (bp)	2,044,111	2,043,929	1,991,399	1,980,172	2,085,679	2,107,472
G+C content (%)	42,2	42.2	42.1	42	42.2	42.1
Predicted CDS	1,954	2,026	1,525	1,525	1,840	1,990
Number of rRNA genes	15	15	8,	7	15	15
Number of tRNA genes	57	57	50	40	57	56

PMC202: https://www.ncbi.nlm.nih.gov/assembly/GCF_019448175.1 (PRJNA750221)

PMC48: https://www.ncbi.nlm.nih.gov/assembly/GCF_011604585.1

K3: https://www.ncbi.nlm.nih.gov/assembly/GCF_001294765.1

S1: https://www.ncbi.nlm.nih.gov/assembly/GCF_001461015.1

JQII-5: https://www.ncbi.nlm.nih.gov/assembly/GCF_006770685.1

HN9: https://www.ncbi.nlm.nih.gov/assembly/GCF_014906145.1

**Table 3:** Clinical signs and mortality in a two-week oral toxicity study using mice.

Group	No. of animals	Clinical sign	Days after dosing													

			1	2	3	4	5	6	7	8	9	10	11	12	13	14
Control	6	Loss weight (15%)	0	0	0	0	0	0	0	0	0	0	0	0	0	0
		No observable abnormality	0	0	0	0	0	0	0	0	0	0	0	0	0	0
		Death	0	0	0	0	0	0	0	0	0	0	0	0	0	0
PMC202 (1.2×10^8^ CFU/ 200 μl/mouse/day)	6	Loss weight (15%)	0	0	0	0	0	0	0	0	0	0	0	0	0	0
		No observable abnormality	0	0	0	0	0	0	0	0	0	0	0	0	0	0
		Death	0	0	0	0	0	0	0	0	0	0	0	0	0	0

PMC202, Freshly cultured live PMC202 strain not subjected to heat or mechanical lysis.
